# In this month’s Bulletin

**DOI:** 10.2471/BLT.21.000521

**Published:** 2021-05-01

In the editorial section, Vageesh Jain and Sam Tweed (322) argue that consensus is needed to standardise COVID-19 control objectives. Hussain Abbas Zaidi and Charles D Wells (323) examine the use of digital health technologies to improve adherence to tuberculosis treatment.

In the news section, Lynn Eaton (326–327) reports on initiatives designed to prevent the next pandemic. Helen Rees talks to Gary Humphreys (328–329) about lessons learnt in pandemic prevention and the need to maintain a focus on polio eradication.

## China

### Blueprint for screening international travellers

Zujin Luo et al. (374–380) document the process used to create a designated hospital.

## Kenya

### Ensuring supply-chain continuity during a pandemic

Dan N Tran et al. (388–392) detail a revolving pharmacy model for essential medicines.

## Papua New Guinea

### Transforming the health information system

Alexander Rosewell et al. (381–387) pilot mobile and geographic information technologies.

## Thailand

### Community surveillance for COVID-19

Nayawadee Kaweenuttayanon et al. (393–397) describe the deployment of village health volunteers.

## WHO Western Pacific Region

### Leveraging national disease control plans

Kerri Viney et al. (330–341) examine a regional tuberculosis response framework.

## Global

### Wildlife, environment, biodiversity and climate

Catherine Machalaba et al. (342–350) identify gaps in national pandemic preparedness plans.

### Building epidemiology emergency response teams

Amy Elizabeth Parry et al. (351–358) examine what is needed to achieve collective competence.

### Adolescents’ sexual and reproductive health 

Rachel Vanderkruik et al. (359–373) review the evidence for mental health outcomes.

### Setting research priorities

Alessandro Cassini et al. (398–401) call for more research on the epidemiology of sepsis.

### Research capacity to support migrants’ rights

Vanessa Brizuela et al. (402–404) describe the need for local evidence in humanitarian settings.

